# A conceptual modeling framework for discrete event simulation using hierarchical control structures

**DOI:** 10.1016/j.simpat.2015.04.004

**Published:** 2015-08

**Authors:** N. Furian, M. O’Sullivan, C. Walker, S. Vössner, D. Neubacher

**Affiliations:** a Department of Engineering Science, University of Auckland, 70 Symonds Street, Auckland, New Zealand; b Institute of Engineering and Business Informatics, University of Technology Graz, 8010 Graz, Austria

**Keywords:** Discrete event simulation, Conceptual modeling, System control

## Abstract

Conceptual Modeling (CM) is a fundamental step in a simulation project. Nevertheless, it is only recently that structured approaches towards the definition and formulation of conceptual models have gained importance in the Discrete Event Simulation (DES) community. As a consequence, frameworks and guidelines for applying CM to DES have emerged and discussion of CM for DES is increasing. However, both the organization of model-components and the identification of behavior and system control from standard CM approaches have shortcomings that limit CM’s applicability to DES. Therefore, we discuss the different aspects of previous CM frameworks and identify their limitations. Further, we present the Hierarchical Control Conceptual Modeling framework that pays more attention to the identification of a models’ system behavior, control policies and dispatching routines and their structured representation within a conceptual model. The framework guides the user step-by-step through the modeling process and is illustrated by a worked example.

## 1 Introduction

Conceptual Modeling (CM) is one of the most important aspects of a simulation project (see [24]). It involves the abstraction of a model from the real world system, identifying what has to be modeled and how. Despite its importance, CM has only recently gained popularity in Discrete Event Simulation (DES) literature. As a consequence, the definition of CM is still evolving and is interpreted slightly differently by varying authors. However, there is a common agreement that it should be independent of any implementation paradigm, or software solution, and that it is initiated early in the simulation project life-cycle and iteratively revisited. According to Pace [20], a conceptual model is a tool of communication between all parties in a simulation project. In addition, among other features, it provides the basis of the model documentation; guides the development of a computer model; provides guidance for experiments; and is an aid for model verification and validation (see [25]). On the other hand, Balci and Ormsby [4] and Balci et al. [5] discuss CM for large scale models and therefore address re-usability of simulation models as the largest gain from structured and standardized CM in addition to the previous features.

A recent summary on definitions, frameworks, representations and other aspects of CM was published by Robinson et al. [27]. Robinson [29] gives a brief tutorial on CM. Issues and further research requirements for CM were summarized by Robinson [24]. Among others, Robinson [24] identifies the following key research requirements: developing consensus over the definition of CM; the identification of requirements of CM; developing methods for designing conceptual models; the definition of modeling frameworks; and moving towards standard methods for representing and communicating conceptual models.

This paper proposes a new framework for CM and hence addresses mainly the design and definition aspects of the research requirements discussed in Robinson [24]. The concepts of the framework are based on previous definitions and frameworks for CM, but replace queuing networks by both more sophisticated mechanisms for entity behavior that enable explicit, centralized control policies (such as dispatching). Control mechanisms (such as optimized dispatch) with complexity beyond common queuing methods have been identified as the key factor for accurately representing a real system, especially for health care models (see [8]). Hence the prevailing practice of representing DES models with queuing networks may not always be appropriate. The paper is structured as follows: Section 2 revisits the most common definitions and views on CM and discusses their relevance for this paper; Section 3 includes a summary and discussion of frameworks for CM and representation methodologies; Section 4 introduces the proposed framework and the new concepts are illustrated by a example from Birta and Arbez [6]; the paper concludes in Section 5 with final remarks and further research topics.

## 2 Definition of a conceptual model

As mentioned in the introduction, to date no generally accepted definition of a conceptual model and the associated modeling tasks exists. This section gives a brief overview of existing literature and concludes with the authors’ view on CM.

Zeigler [35] is the first work that pays attention to different abstraction levels of simulation models by identifying five elements: the real system; the experimental frame; the base model; the lumped model; and the computer model. The experimental frame denotes the circumstances under which the real system is observed (input & output patterns). The base model is a hypothetical, complete representation of the real world, which is obviously not known. The lumped model is a simplified version of the base model that is able to reflect all input & output patterns and the computer model denotes the implementation of the lumped model. In the terminology from Zeigler [35], the lumped model is mainly independent to the computer model and represents what is now understood as a conceptual model.

Balci [3] analyses the early stages of a simulation study and identifies iteratively repeated steps that include model formulation, model representation and programming. Nance [14] distinguishes between the model that exists in the mind of the modeler, the conceptual model, and its representation for use in for communication, the communicative model. In the defense sector, Pace [17], [19] interpret the conceptual model as a definition of what has to be modeled and how, providing information on assumptions, algorithms, characteristics, relationships and data. Further, Lacy et al. [12] distinguishs between a domain-oriented problem that describes the requirements of the model and a design-oriented model that is used as a basis for model implementation.

More recently, Balci and Ormsby [4] discuss CM for large scale simulations. Thereby, they identified three major abstraction layers that a simulation model passes through in it’s life cycle: simulation conceptual model; simulation design model; and simulation model implementation. They argue that the implementation is accomplished by using programming languages as Java, C++ or C#. The transition between the design layer and the implementation layer is undertaken by the use of discrete-event world views, e.g. event scheduling, three-phase approach or process interaction (see for example [2]). Hence, the design model is a description of the components of the implementation model using object-oriented or procedural paradigms. The conceptual model represents the highest layer of abstraction and is the model that is formulated in the mind of the modeler. Note that their work is motivated by large scale models in the defense sector. The number of modelers and domain experts involved in these studies is usually large. Hence, requirements for CM differ to business oriented models by focusing on documentation, communication and re-usability (see [25]).

In Birta and Arbez [6] state that CM is concerned with developing a meaningful representation of the real system. Further, they define two major requirements for a conceptual model: it must be sufficiently transparent to be used as a means for discussions by all stakeholders in the project; and it must be sufficiently transparent so that it can serve as a specification for the computer program.

In Karagöz and Demirörs [10] state that CM is a tool that provides a clear understanding of the target domain or problem. Hence, it is a simplified representation of the real system including structural and behavioral features.

The most recognized definition of a conceptual model is given in Robinson [25]. Robinson argues that a conceptual model is derived from an understanding of the problem situation, which is external to the conceptual model. Hence, it is a partial description of the real world that is sufficient to address the problem situation. Further, it consists of four main components: objectives; inputs (experimental factors); outputs (responses); and the model content. Content is further divided into the scope of the model and the level of detail. During the identification of the model’s content assumptions and simplifications have to be made. This leads to the following definition of a conceptual model (see [25]):

“The conceptual model is a non-software specific description of the computer simulation model (that will be, is or has been developed), describing the objectives, inputs, outputs, content, assumptions and simplifications of the model”.

The task of conceptual modeling is therefore concerned with an understanding of the problem situation and the identification and determination of the components of a conceptual model. Furthermore, in Robinson [28] the model design is added as an artifact to the project life-cycle. The model design is defined as the design of constructs for the computer model, see also Fishwick [7].

Note that the definition in Robinson [25] and Balci and Ormsby [4] are motivated by varying scales and problem domains. While Balci and Ormsby [4] is based on large scale military applications that usually involve hundreds of developers and domain experts, from scratch coding and long project life cycles, Robinson [25] is intended for business-oriented models. These models usually have a rather short development time, are built using visual commercial software tools and re-usability is not a major concern, see Pidd [22]. Hence, Robinson [25] is more concerned about the abstraction of a real system and components involved, where the view of Balci and Ormsby [4] has a more descriptive nature.

The consensus is that a conceptual model should be software independent and that a clear distinction between CM and model design has to be made. The ideas and frameworks proposed in this paper are based on the definitions in Robinson [25], as the authors are usually involved in business-oriented simulation projects. However, during the discussion of existing frameworks for CM that follows in Section 3, it will become apparent that we believe that a model’s content is not sufficiently represented by existing approaches. Moreover, we agree that a clear separation between conceptual models and design models should always be maintained to avoid the modeling being driven by implementation paradigms, such as DES world views.

## 3 Conceptual modeling frameworks and representation methodologies

In this section we will discuss existing frameworks for CM and identify some potential improvements. Further, we revisit notations and techniques for conceptual model representations.

### 3.1 Conceptual modeling frameworks

The first structured guidelines for the CM process were provided by Shannon [30]. Shannon identifies four major steps: specification of the model’s purpose; component specification; parameter and variable specification; and the specification of the relationship between variables, components and parameters. Nance [14] introduces the conical method, an object oriented hierarchical method for developing the model definition (scope) in a top-down manner and the model specification (or detail) in a bottom-up manner. Pace [17], [18] explore four stages for CM development (similar to Shannon [30]), including: collection of information; identification of entities and processes; identification of simulation elements; identification of relationships between simulation elements. Nance and Arthur [15] adopt software requirements engineering for CM. Van Der Zee and Van Der Vorst [33] and van der Zee [32] propose a framework for manufacturing systems with a strong emphasis on simulation control. Based on the definitions in Section 2 [4] introduces a CM framework for large scale simulation models.

More recently, Karagöz and Demirörs [10] published a classification and comparison of CM frameworks (mostly in the defense sector). Further, they introduce KAMA, a UML based methodology for CM. Tako et al. [31] present Parti-Sim that guides the modeler through interaction with stakeholders and domain experts in the CM phase. Kotiadis and Robinson [11] use Soft System Methodologies (SSM) for the knowledge acquisition and model abstraction tasks.

Two frameworks, one by Robinson [26] and ABCmod from Birta and Arbez [6], are of particular interest for this paper and hence are explained in more detail in the sequel.

#### 3.1.1 The Robinson framework

The framework of Robinson [26], referred to as the Robinson framework for the remainder of the paper, consists of five major steps, as outlined in the remainder of this subsection.

The first step is concerned with an understanding of the problem situation. In most simulation studies the problem situation is not entirely known or understood. As a consequence assumptions have to be made and documented. SSM, a formal structuring method for this step, is suggested.

The second step deals with the determination of the objectives. According to Robinson [26] they drive all aspects of the modeling process and are a subset of an organization’s aims. Further, objectives can be classified into modeling and general objectives, where the latter are concerned with the flexibility, run-speed, visual-display and model/component reuse.

The identification of the model’s input and output make up steps three and four. Outputs can be used to determine if objectives have been met and, if not, why not. In Robinson [26] it is argued that consideration should be given to how the output is reported (e.g. statistical values or graphs). Input factors represent the model data that can be changed in order to achieve the modeling objectives.

The last step, and, in our opinion, the most important one, deals with the determination of the model’s content. It is further divided into the identification of the model’s scope and it’s level of detail. A crucial assumption, as will be argued later, is that any simulation model can be conceived in terms of four types of components: entities; activities; queues; and resources. Based on this assumption, determining a model’s scope can be accomplished in three steps: identification of the model boundary; identification of all components within the boundary; decisions on inclusion/exclusion of all components identified. Determining the level of detail is concerned with the amount of detail to include for each component in the model. All steps are reported in tabular form, including details of entities (e.g. arrival pattern, attributes and routing), activities (e.g. attributes, entity routing and duration), queues (e.g. maximum length, selection routine and routing) and resources (e.g. where is it required, shifts and skill levels).

During all steps assumptions and simplifications have to be made in order to achieve the simplest model that still reflects all input/output patterns. In Robinson [26] it is suggested that these are documented and assessed, to determine the level of impact they have on the model responses and the confidence in them.

#### 3.1.2 ABCmod

The ABCmod framework was first published in Birta and Arbez [6] and in a revised form in Robinson [27]. It is closely related to the three-phase world view for DES, see [21] or [2]. It consists of two main building block categories, entity structures and behavior constructs. Entity structures define the specification of entity instances in a model. Four major roles can be identified: consumers; resources; groups; and queues. It is noted in Birta and Arbez [6] that those roles may be ambiguous for some entities.

Behavioral constructs are classified into activities and actions. An activity is an indivisible unit that characterizes an interaction among entities, is associated with a purposeful task and evolves over a nonzero (but usually finite) interval of time. It has four major components: a starting condition; a list of state changes upon start; a duration; and a list of state changes upon termination. Activities can be classified as: scheduled (happen at defined point in time); conditional (occur upon the satisfaction of a pre-condition); and triggered (initiated in the state changes of another behavioral artifact). Actions denote singular state changes in time, similar to common events.

One important feature of the ABCmod framework is that it makes a clear distinction between structural and behavioral constructs. ABCmod models are divided into a high level model and a detailed model.

The high level model consists of: a structural overview (brief description of entity structures); a list of data modules; and a behavioral overview (a list of behavioral constructs and a collection of activity cycle diagrams).

The detailed formulation is organized in five sections: structural components (detailed specification of all entity structures); data components; input components; output components; and behavioral components (tabular representation of all behavioral artifacts, their attributes, conditions and state change routines).

Although, Arbez and Birta [1] argue that the ABCmod framework is independent from implementation paradigms (in particular Birta and Arbez [6] demonstrates how an ABCmod model can be transformed to any world view) it is more rigid than the Robinson framework. It is closely related to the three-phase world view for DES and incorporates concepts of the latter for the description of a conceptual model (in particular pre-conditions of activities and state change routines). It is unclear whether to place ABCmod within the domain of CM frameworks or model design frameworks.

#### 3.1.3 Discussion of existing frameworks and motivation

Both ABCmod and Robinson’s framework, discussed in Section 3, are based on the assumption that most DES models can be represented as queuing systems with tight resource-queue couplings, see for example [23]. However, in Van Der Zee and Van Der Vorst [33] the need for more sophisticated control structures for manufacturing models is expressed. Furthermore, in certain applications, such as in the health care sector, a large proportion of resources are human beings. As humans will often perform a variety of tasks and can be assigned to tasks in many different ways, capturing system behavior often requires more complex dispatching (of resources – e.g., humans – to entities/consumers – e.g., tasks) and control policies (e.g. staff workload balancing), as argued in Hay et al. [8]. Table 1 provides a summarized assessment of the two frameworks introduced in this section with regards to dispatching, control and other features.

ABCmod [6] pays more attention to the distinction between structural and behavioral components of a conceptual model than Robinson [26]. Furthermore, Birta and Arbez [6]’s framework consists of designated steps for visualizing entity flows, while Robinson [26] only suggests to do so without reserving defined steps in the framework. Furthermore, role changes (e.g. resources acting as entities/consumers during certain activities) are not considered in Robinson [26].

Both frameworks are proposed for the design of queuing models. In applications where not all entities form queues in which they wait to begin activities such an approach may not be the best modeling choice. In particular, policies and decision-making on a scale beyond queuing mechanisms should be captured explicitly in a conceptual model.

However, CM modeling is concerned with guiding the modeler through the process of defining and documenting model boundaries, components, assumptions and simplifications in a structured way (according to Robinson [25]). This is not only true for components and individual behavior (e.g. processes and entities), but also for the rules nested at a system level, e.g. dispatching. Especially in health care systems, the identification of how patients are treated and how jobs are dispatched is the most crucial factor for an accurate representation of the system (see [8]). Thus, it seems unsatisfactory that existing frameworks do not provide any explicit modeling concepts for capturing the system’s behavior on a global level (such as dispatching and other control policies).

In this paper we extend current CM frameworks by introducing *control units* as part of a conceptual model’s components. These control units represent the rules governing entity flow within each sub-area of the system. Their definition requires the same care and sequence of steps as other components (boundaries, scope, level of detail, assumptions and simplifications). Thereby, the modeler is guided through the steps of identifying and reporting system rules. Furthermore, the removal of the explicit use of queuing structures renders role classification (resource/consumer) redundant. Especially for health care models, the most natural way to represent a system is not necessarily a queuing structure. Staff members engage in a variety of activities (e.g. teaching or meetings), act as a consumer and a resource at the same time (e.g. inexperienced clinicians assisting experts in certain tasks) and are not rigidly associated with serving queues for certain tasks. Abstracting control of entity flow to provide not only queuing mechanisms, but more sophisticated control structures enables elegant CM development for these systems.

The authors are currently involved in a simulation study on a pathology lab where the main task of clinicians is to perform analysis and reporting tasks on samples. In addition, the clinicians engage in numerous other tasks such as teaching, various meetings with different attendance requirements, and emergency or scheduled call-outs to other facilities. Dispatching of tasks is, hence, not only dependent on samples in the “queue” and their priority, but many other factors that affect task assignment decisions. In particular, roles of clinicians are not always unambiguously defined. Especially for teaching (both classical classroom teaching and assistance during analysis, in order to gain experience) and meeting activities, it is not clear if clinicians actually serve a queue, or are queued up themselves.

In another study we assess the possible improvements from applying automated optimization-based job dispatching policies for orderlies performing patient transits from wards to diagnostic departments and vice versa. All transits requested at certain points during simulation are transformed to a mixed integer program that is solved to optimality and serves as a basis for dispatching decisions. Results are compared to policies currently in place to identify possible improvements. Both models may not be best represented as classical queuing systems without cumbersome adaptations and extra definitions. Hence, it seems inconvenient to formulate conceptual models based on that paradigm. Thus, a more general representation for capturing dynamics in a conceptual model is beneficial. It also enables the integration of more sophisticated dispatching strategies, e.g. optimization routines, in a well-defined conceptual way.

### 3.2 Conceptual modeling notations and representations

As pointed out by Onggo [16], the main challenge in designing representations for CM is to devise a representation that can be understood by all stakeholders. A list of some possibilities is provided by Wang and Brooks [34] and includes textual representation, process flow diagrams, logic diagrams, activity cycle diagrams and UML. Liston et al. [13] discuss the possibility to use SysML as a representation support for conceptual modeling. SysML is general-purpose graphical modeling language that has been designed for systems engineering and is based on UML. In total it consists of eight diagram types that are divided into structural and behavioral diagrams. Although, it has gained some popularity (especially in industry), it’s weaknesses are that it involves a rather steep learning curve requiring extensive training, and that diagram types are interpreted and used differently among modelers.

As most modelers have their method and software of choice to draw representative diagrams, we do not propose the use of a specific package (e.g. UML or SysML). However, throughout, we will outline possibilities for the more inexperienced practitioner.

## 4 The conceptual modeling framework

In Section 3.1.3 some shortcomings of existing frameworks for CM were discussed. Thereby, the need for more attention to model control concepts was motivated. The framework presented in this section, Hierarchical Control Conceptual Modeling (HCCM), breaks with the assumption that all DES models are best represented by queuing systems. Therefore, we introduce control structures that add more flexibility in the abstraction and conceptual modeling process. Both concepts and components are illustrated using a working example from Birta and Arbez [6].

### 4.1 Port problem – A working example

To illustrate the concepts of the HCCM framework we will use an extended version of the tanker port problem introduced by Birta and Arbez [6]. In the original problem tankers arrive at a harbor site with respect to an arrival process, where they wait to be towed to a loading area by a tugboat. In the loading area they are berthed by the tugboat and then filled with oil. After the loading process, they are de-berthed and towed to the harbor, also by tugboats, as soon as tugboats are available, where they leave the model. Both berthing and de-berthing, are performed in a first-in-first-out (FIFO) way according to the arriving and loading completion times of tankers. The berthing area holds a certain number of actual berthing sites. The number of berthing sites is an experimental parameter (input factor) that can vary across different scenarios to be evaluated.

To demonstrate the features of the framework proposed in this paper, the problem in Birta and Arbez [6] is extended by adding some new structures and behaviors. On the other hand, some aspects (for example, occurrences of storms and the termination of moving activities for tugboats) are omitted for space and simplicity reasons.

First, a second (container) port for container ships is introduced. It performs analogously to the tanker port, and also controls a single tugboat. Second, both tugboats have to be re-fueled when their fuel level reaches a lower limit. Third, if a single port is empty, the port offers to send the tugboat to assist the other port. In the remainder of this subsection an informal description of the behavior of entities in the model is given.

Tugboats can move between the harbor area and berthing area in the same port with no ship in tow (“moving empty”) under the following conditions:

• A tugboat moves from a berthing area to the harbor area in the same port if:
  1. no ship is ready for de-berthing;
  2. at least one ship is waiting in the harbor; and
  3. a berthing site is available.
• A tugboat moves from the harbor to the berthing area in the same port if:
  1. no ships are waiting in the harbor; and
  2. at least one ship is loading or waiting for de-berthing.
• If both tugboats are working in the same port tugboats are not allowed to move without a ship in tow. This condition assumes that arrivals and loading completions are somewhat balanced, so the two tugboats will be spread evenly “enough” between the harbor and berthing area while performing towing duties. It also keeps the rules for controlling tugboats at a degree of complexity that is not beyond the scope of this paper.

A port reports that it is idle if no ships are present and the tugboat is waiting in the harbor area. The tugboat is sent to the other port if at least three ships are waiting in the other harbor area. Tugboats can only move between the harbor areas of the two ports. Further, a port requests a tugboat back if a ship arrives and waits in the harbor area. If the fuel level of a tugboat reaches a lower limit the tugboat moves to the tugboat service station where it is served according to the FIFO principle. After completion of the refueling service, the tugboat is sent to the port with less tugboats unless the port with less tugboats is empty and the other port is not.

### 4.2 Behavior revisited

Before presenting the actual steps of the HCCM framework some common behavioral components of DES models, activities and events, are revisited and their relevance for conceptual modeling are discussed.

While activity based modeling may have it’s limitations regarding simulation implementation, CM is able to benefit from an activity based view of behavior. Heath and Brailsford [9] argue that events and activities are the most natural way to describe the behavior of many platforms. In particular, humans engage in a certain activity until finished or interrupted and then proceed with the next one. In other words, an activity encapsulates a unit of behavior that has been identified as having relevance from the perspective of the project goals (see [6]).

In general we agree with the definition of activities provided by Birta and Arbez [6] (see Section 3.1.2) that an activity is an indivisible unit that characterizes an interaction among entities, serves a purposeful task and stretches over a non-zero amount of time. However, we do not require at least one resource being part of every activity, e.g., a patient filling out forms. On the other hand, events (or actions by Birta and Arbez [6]) are instant changes of the model’s state, for example, the arrival of a tanker.

In the classical literature, events and activities are divided into scheduled, conditional and sequential events/activities depending on their trigger mechanism. Although, this is closely related to simulation implementation it has also relevance for CM. During the abstraction process that leads to a conceptual model, the modeler has to identify under which circumstances behavior may take place (i.e., at the start or end of an activity) and whether they take place at fixed points in time (scheduled), or with regard to decisions or satisfaction of conditions (conditional), or subsequent to previous behavior (sequential).

While scheduled and sequential behavior is defined in the classical way in the HCCM framework, conditional behavior requires more detailed consideration. In the port problem tankers are berthed if a tugboat and a berthing site are available. However, the behavior of the tanker (wait, berth, load, de-berth and leave) triggers the request for the start of this activity. On the other hand, the tugboat moves between a harbor and a berthing area without a tanker in tow dependent on rule sets defined in the model’s control structure, independently of the tugboat’s behavioral path. Both are normally considered conditional activities, as their start events are conditional. However, their motivation differs significantly. In the first case the tugboat moves depending on its behavioral path and the subsequent request from a tanker entity but, in the second case, it moves in response to pre-defined rule sets that are part of the model’s control structure rather than reacting to an individual entity’s behavioral path.

Therefore, we split conditional behavior into two subclasses. Furthermore, as it is the intention of the HCCM framework to centralize rules and conditions to control units they are labelled controlled behavior, rather than conditional behavior.

Requests for events or activities are filed by an entity (or a group of entities), based on its current state, previous history of states and behavioral path (often called a process) in the model. These requests may require preliminary or concluding activities, for example, a tugboat may need to travel from the harbor to the berthing area to tow a tanker that has completed loading and requested a de-berthing. However, the exact composition of the preliminary and/or concluding activities is determined by the model’s control structure, so only the actual requested activity is classified as requested. The preliminary and/or concluding activities are classified as system activities because the control structure of the system determines when those activities take place.

**Fig. 1:**
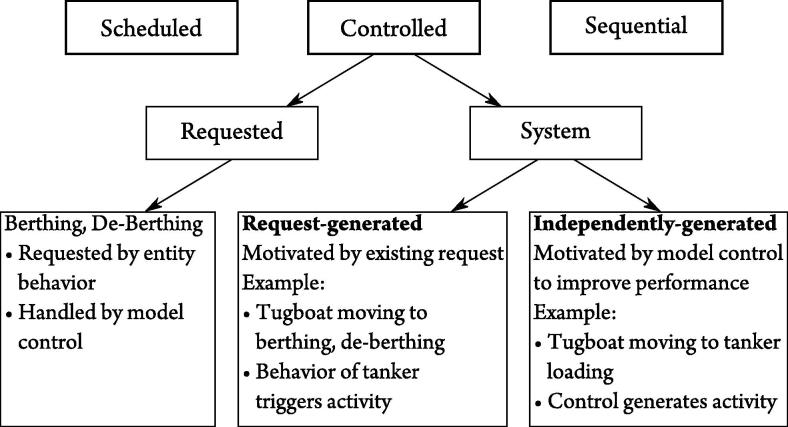
Classification of behavior.

System behavior is further classified as either request-generated or independently-generated, as illustrated by Fig. 1.

### 4.3 The structure of the HCCM framework

**Fig. 2:**
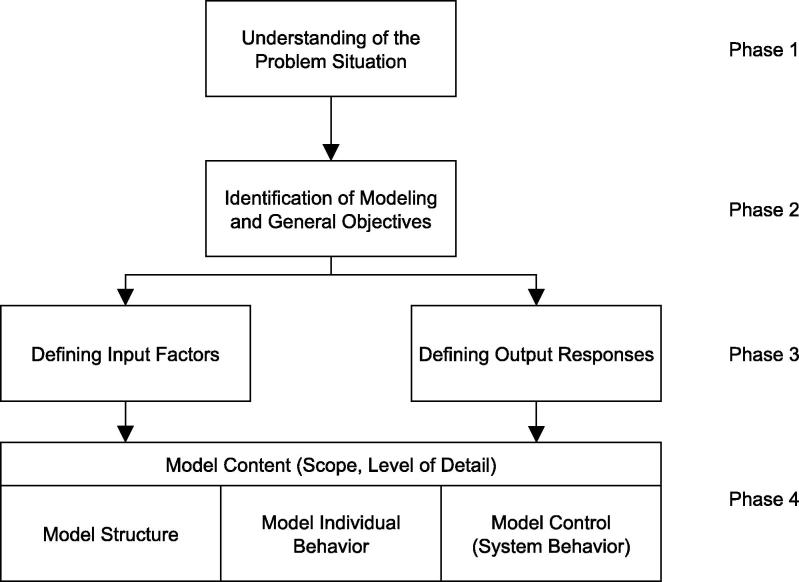
Structure of the HCCM framework.

The steps of the HCCM framework include steps from the framework by Robinson [26], as well as the separation of structural and behavioral components from within ABCmod (see [6]). Further, the model content is extended by control structures and associated rules. The overall steps of defining a HCCM model are illustrated by Fig. 2, which is based on definitions by Robinson [26].

As modeling is naturally an iterative process, phases (1)–(4) are likely to revisited during the modeling task.

In the following all steps are outlined in detail. A full description of the resulting conceptual model is provided in Appendix A.

#### 4.3.1 Understanding the problem situation

The first step in the CM modeling process is devoted to the understanding of the problem situation. As pointed out in Robinson [26], several difficulties may arise during this task: the problem situation is either not understood or expressed clearly; or domain experts, modelers and clients may have different views on the behavior of the system. In either scenario, Robinson [26] suggests that “speaking with the right people and asking searching questions is vital to developing this understanding. The modeler should also be willing to suggest alternative interpretations with a view to unearthing new ways of perceiving the problem situation”. Further, formal problem structuring methods, such as SSM, may be used (see [11] or [31]).

The intended result of this step is an informal, textual description of the problem situation, such as the description of the port problem in Section 4.1. Assumptions that have been made in the gathering of the problem situation should be well documented and included in the problem description.

#### 4.3.2 Identification of objectives

Robinson [25] distinguishes between two kinds of objectives, general objectives and modeling objectives. General objectives denote requirements on the simulation model as a tool, e.g. run-time, development effort flexibility to changes, visualization requirements or re-usability. Those are most likely determined by the nature of the project, but should always be kept in mind when choosing the level of detail and complexity of the model.

Modeling objectives are a subset of the overall aims of the organization [26]. Further, Robinson [26] states that “the modeling objectives should always be expressed in terms of what can be achieved from the development and use of the model”. The modeling objectives for the port problem could include the determination of required berthing areas for tankers and container ships to keep the 90% quantile of throughput times under a certain limit. General objectives may include a development time of two months, reasonable run-times (e.g. 10 s) and a visualization for presentation to the stakeholders.

#### 4.3.3 Outputs

Outputs (responses) can be used to assess whether the modeling objectives have been met or to point out the reasons why not, if they are not met [26]. Outputs can have the form of single aggregated numerical values (e.g., mean, minimum, maximum, variance or confidence intervals), or streamed data (e.g., in the form of a time series). Consideration has to be given to form (tabular or graphical) representation of responses. Outputs for the port problem could include throughput times of tanker and container ships that allow the assessment of the model’s objective.

#### 4.3.4 Input factors

Input values, or experimental factors, is usually data that can change over different experiments and simulation runs. Their definition includes the identification of their range and how they are included in the model. For the port problem the inputs denote the number of berthing areas for either port, and the threshold above which tugboats are requested for assistance.

Note that, especially in health care models, input factors may not always denote single measures, but also include policies. For example, in a patient transportation model currently under development by the authors, the goal is to assess different dispatching strategies of orderlies to transit tasks. These include: choose the closest available; select the orderly that is able to be at pickup first (could still be performing another task, hence not necessarily available); or assignment by a dispatching optimization algorithm. Those policies can still be seen as input factors for experiments, but also require a distinct tool-set for integration in the conceptual model, see Section 4.3.7.

#### 4.3.5 The model structure

The model structure is defined by the entity structures that are included. Entity structures define the specification of entity instances (i.e. which state variables are associated with them) in the model, similar to classes and instances within the object-oriented software paradigm.

Entities and their aggregations are the core structural elements of any DES model. As illustrated by Table 1 existing frameworks include similar roles and aggregation concepts. However, in HCCM no explicit distinction between resource and consuming roles is made. Further, common aggregation components, such as queues and groups are summarized by control structures, as will be explained later.

The HCCM framework consists only of two types of entity structures: active and passive. Despite the impression that this classification is closely related to the resource/consumer paradigm, its intention is different. Active entities have a particular behavior associated with them. This class consists of resources, consumers and entities that change their role. In the working example of this paper, both tankers and tugboats would be active entities. Tankers and tugboats engage in certain activities that are defined by behavioral structures. On the other hand, passive entities are not associated with a behavioral flow, for example, the harbor waiting area or even the berthing area.

**Fig. 3:**
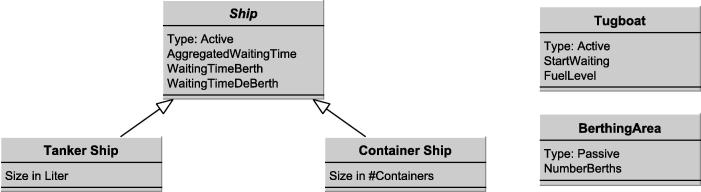
Entity structures.

After an assessment of all entities in the system the modeler has to determine which to include in the model (defining the scope of the model as in Robinson [26]) and how they are specified (defining the level of detail as in Robinson [26]). This can either be done in tabular form or using UML class diagrams, as illustrated by Fig. 3. Note that definitions for harbor areas and re-fueling stations have been omitted due to space reasons.

Furthermore, we propose modelers may capture the system in an informal graphical way, either based on logical or physical components, similar to the structural view of Birta and Arbez [6]. Thereby, entities with fixed locations outline the structure of the model. Additionally, moving entities should illustrate the flow of the model. It is intended to be a more informal way to draw a picture of what is happening and where it is happening in the model. It is up to the modeler to determine their preferred way/style to illustrate entities and their flows. A general blueprint of the system is often helpful to gain an agreed understanding of the model among all stakeholders and is hence encouraged in the CM process.

**Fig. 4:**
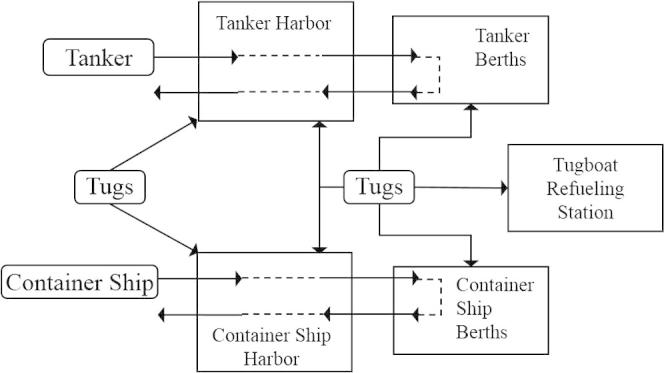
Structural view of the port problem.

Fig. 4 gives a possible structural view of the port problem.

#### 4.3.6 Individual model behavior

In the next step the entities’ individual behavior has to be identified and reported. Decisions have to be made regarding what behavioral artifacts are included and in what detail they are represented in the model. For example, we included refueling activities of tugboats. There might be a scenario where this is not within the model’s boundary, or no data is available and hence it is omitted from the model. Such an exclusion would, according to Robinson [26] define the model’s scope. Further, we simplified the berthing times of ships to be independent of their size. Such simplifications define the level of detail included in the model.

**Fig. 5:**
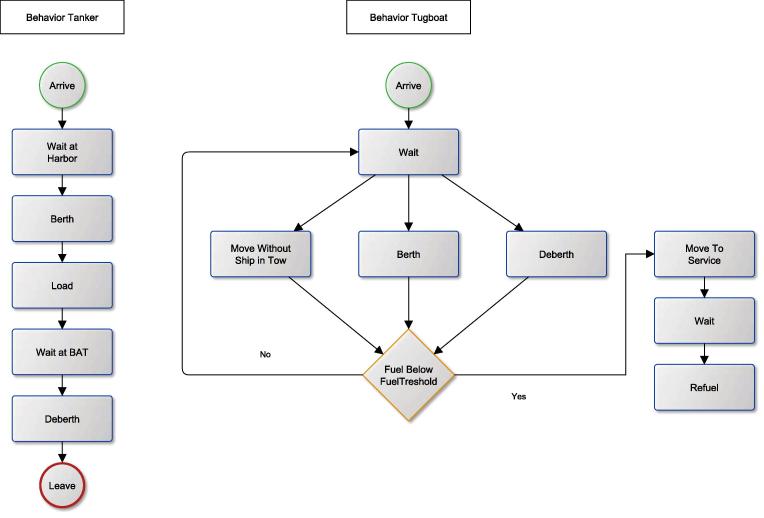
Behavioral view port problem.

We suggest a visual representation of how entities flow through the system, similar to the behavioral view of Birta and Arbez [6] that uses activity cycle diagrams. Note that there are several other possibilities to capture sequences of behavior, including (but not limited to): UML and SysML activity diagrams, state charts or sequence diagrams; business process diagrams; flow charts; and event graphs. We do not suggest the use of a specific package or software, but illustrate the behavioral flow of tanker ships and tugboats using business process diagrams in Fig. 5.

Further, the detail included in activities should be reported, e.g., in tabular form. Thereby it is important to identify their type (see Section 4.2), attributes, participating entities, state changes and the specification of requests made for that activity. For example, when is the activity requested (under which circumstances), what time is a request made, are there blocked resources, and/or what the priority levels should be, and so on.

Explicitly defining attributes within requests provides more flexibility in the definition of control structures as decisions may be based on a wider range of information. Note, that request attributes might even change over time, e.g. accumulating priorities of patients.

Furthermore, it should be documented whether activities can be preempted or interrupted and if so what consequences that implies. An example is given by Table 2.

#### 4.3.7 System behavior

Thus far the only true extension to existing definitions and elements of DES CM described in this paper is the extended classification of events and activities. Even if that might help the understanding of the real world and its representation, it does not yet contribute to the advanced control representation of the model proposed here. The control view, described in this subsection, adds the control structure required for advanced control mechanisms to a conceptual model for DES.

The key elements of the control view of a HCCM model are control units, activities and events. Together they form a hierarchical tree structure, where activities and events are represented by leaves and control units are represented by internal nodes. The hierarchical structure of control not only units delimitate the areas of responsibility (e.g. container and tanker ports), but also enables different levels of decision making, e.g. decisions on staff or resource balancing are made higher up in the tree structure. The representation of activities and events as leaves identifies the behavior that can be handled/triggered by the control unit associated with the corresponding parent node.

Control units include a set of rules that determine the conditional behavior of the model. Note that the rule set can take many different forms and may also include optimization strategies that determine next events and activities triggered in the model. Further, they may also hold a set of attributes and state variables, capturing the system’s specification and changing state over time.

The depth of the designed tree is a modeling choice, which should be made with great care. Too much granularity leads to unnecessarily complex models that are cumbersome to deal with. Whereas too few control structures also can lead to rising complexity as conditions, dispatching and interactions get harder to handle within each control unit.

One significant feature of control units is that they replace common entity aggregation structures such as queues and groups. Standard queuing networks are not able to elegantly capture the behavior of entities that act as resources for many different tasks, and also become consumers in the same model. Dispatching rules have to be defined that consider the state of all queues associated with the entity in (possibly) different roles. As a consequence queues no longer act independently and handling their interaction can become cumbersome.

Although the port model used in this paper is relatively simple and straight forward, advantages of a non-queuing based control description can be observed. First, tugboats change their roles between providing (berthing and de-berthing) and consuming (re-fueling) resources in the model. Second, within one port area (tanker or container ship) their actions are not only triggered by classical queue-selection policies, but also in anticipation of future resource requirements (empty moves), which is classified as “system generated behavior” according to Section 1. Third, the triggering of tugboat moves between two ports is also not motivated by selection policies. It is based on the status of multiple entities and entity aggregation constructs (tugboats, berthing and harbor areas) distributed over the entire model. Hence, a centralized description of policies seems to be a more natural approach than distributed selection policies and routing conditions.

Informally speaking, control units handle pools of entities and their requests. Guided by a defined set of rules they make decisions on the temporary engagement of those entities within activities and events. Thus in their simplest versions they may still be seen as standard entity-aggregation concepts like unstructured groups or ordered queues (see [6]), but are also able to reflect more complex scenarios and interaction structures with rather loose entity-task couplings.

Rule sets of control units determine: what requests can be dispatched; how they are dispatched; what system behavior has to be initiated; and what requests are out of date. This allows a centralized definition of system behavior that is not nested within other model components, e.g. queues or activity conditions. Thereby a more structured identification of system behavior is made. Furthermore, due to explicit representation of the strategies, control policies can also be handled as input factors in a more elegant fashion, rather than altering boolean conditions of various activities.

Hence, the last step of the HCCM framework is to identify the system’s control structure and the associated rules, e.g. dispatching, staff workload balancing. The models components (what rules to include) and the level of model detail must be determined, considering questions such as:

• What decisions are made?
• Where are decisions made and by whom?
• On what basis are decisions made (e.g. what state-variables)?
• How are decisions structured (e.g. overruled)?
• What simplifications and assumptions are made?

For example we excluded empty moves of tugboats when the two tugboats operate in the same port. This could either be an assumption because one does not know if they ever move empty in that situation, or a simplification because it is deliberately left out. However, the exact definition of these rules and strategies is an important part of the CM process and should be explicitly dealt with by a framework for CM.

**Fig. 6:**
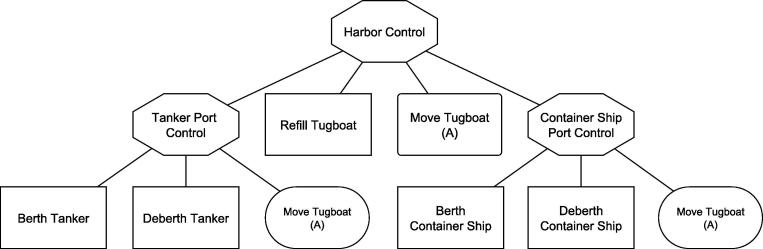
Control view of port problem. Note that parameters A, B and C indicate different empty moves of tugboats. A can occur between the two harbor areas and the service station, whereas B and C denote moves within one port area between berths and harbor area.

The first task is to define the control structure of the model, this includes the identification of control units and their relation. We suggest a tree structure for the organization of control. For the port problem we include control units for either port and the overall harbor. Further, it has to be defined what behavior is controlled (either requested or system) by those units, e.g., for the harbor control unit berthing, de-berthing and tugboat moves are controlled activities, for the port control unit controlled activities are tugboat moves and refueling. We suggest that the resulting treestructure is documented in a graphical form, see for example Fig. 6. Rectangles with cut corners represent control units, normal rectangles represent requested activities and rounded rectangles represent system activities.

Further, a more detailed specification of control units is suggested. That could be either included in the control tree, using representations of control units that are similar to UML class diagrams, or in a separated tabular form, see for example Table 3.

**Fig. 7:**
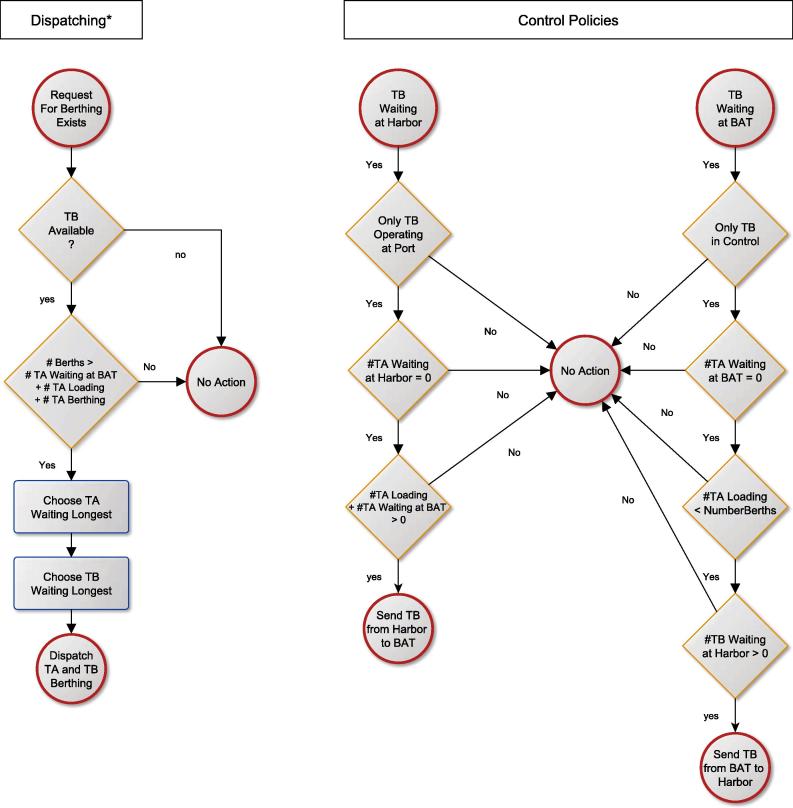
Tanker harbor dispatching and control rules (∗ Only dispatching control rules are shown, but rules for de-berthing can be defined analogously).

**Fig. 8:**
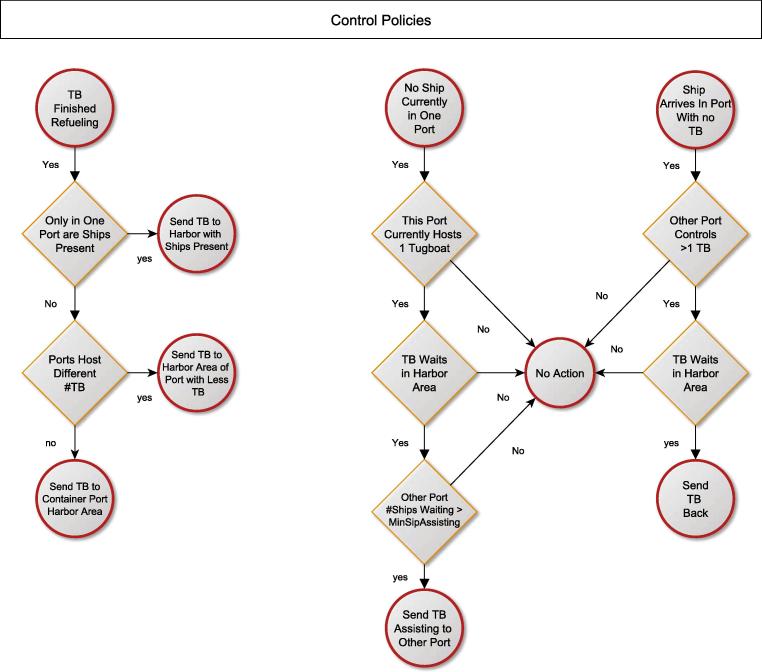
Overall port dispatching and control rules.

Next, the rule sets for control units have to be specified. This can be done either in textual form, pseudo-code or in diagrammatic form. The rule-sets for the tanker harbor are illustrated by Fig. 7, Fig. 8 using logical flow diagrams.

#### 4.3.8 Simplifications and assumptions

Throughout all steps of the HCCM, assumptions and simplifications may be made. We recommend that those are reported and documented in a structured way. Further, it may help to assess the confidence that can be placed in them and their impact on the model, as suggested by Robinson [26]. An example for assumptions and simplifications for the port problem is given in Table 4.

## 5 Conclusion and further research

In this paper we discuss the need for explicit control structures within conceptual models. As conceptual modeling denotes the abstraction process of a real world system to a simplified model, components and individual behavior are not the only concepts that need to be identified and documented. Strategies and control policies, e.g., dispatching, should be explicitly included in any CM definition.

Further, the step-by-step HCCM framework for CM is introduced and illustrated by a working example. HCCM guides the modeler through the most important steps of CM. It facilitates the modeling of more complicated systems where multiple entities interact across various activities. Further, it explicitly provides model control structures responsible for decision making in the system. By centralizing the model’s logic it enables faster and easier adaption to different policies.

The authors are currently using the new framework for a variety of real-world simulation studies that should demonstrate it’s applicability to complex and applied problems. Thereby, also mathematical programming and other optimization techniques will be included in the control policies of simulation models. Further, not only does the conceptual representation of a model benefit from more flexible mechanisms than queuing networks, but these modeling benefits also transfer to the design of models and their implementation. Thus, it may be of interest to transfer the idea of designated control units to model design and implementation in a follow-up paper.

## Figures and Tables

**Table 1:** Comparison of CM frameworks.

Feature	Robinson	ABCmod
Components	**Structure**	**Structure**
	Entities	Consumers
	Queues	Resources
	Resources	Queues
	**Behavior**	Groups
	Activities	**Behavior**
		Activities
		Actions

Structural description	Tabular entity description	Tabular entity structure
		Symbolic toolkit for model blueprint

Separation of structure/behavior	Not explicitly	YES

Activity description	Tabular description of	Tabular description of
	Attributes	Attributes
	State change (including triggering and scheduling of future behavior)	Routing
		Scheduling
	Type	
	Interruption and pre-emption	

Entity description	Tabular description of	Tabular description of
	Attributes	Attributes
	Arrival pattern	
	Routing	

Entity role changes	Not mentioned	Implicit through activity description

Entity flow description	Through routing description of components	Activity cycle diagrams
		Through activity description
	Visualization suggested	

Resource flow description	Not mentioned	Activity cycle diagrams
		Implicitly through activity description

Model control	Implicit through:	Implicitly through:
	Queue selection routines	Activity pre-conditions
	Routing description of components	Queue selection routines

Dispatching	Implicit through	Implicitly through
	Queue selection routines	Activity pre-conditions
	Routing description of components	Queue selection routines

**Table 2:** Berthing activity definition.

Tanker berthing	
Participating entities	Tanker, Tugboat (TB)
Start type	Requested
End type	Scheduled

Start state changes	# TA Berthing += 1;
End state changes	TB.FuelLevel = TB.FuelLevel - FuelCons; # TA Berthing -= 1;

Attributes	Description/value
Duration	20 min
FuelCons	10 l

Request attributes	Description/value
TimeRequest	When was the request made
Request specification	Request is filed at tanker arrival

**Table 3:** Control unit definition.

Name	Entities	Attributes
*Control Unit Definition*		
Tanker Port	Tugboat (TB)	MinShipAssisting = 3
	Tanker Ship	#TA Waiting at BAT
	Berthing Area Tanker (BAT)	#TA Waiting at Harbor
		#TA Loading
		# TA Berthing

Container Port	Tugboat (TB)	MinShipAssisting = 3
	Container Ship (CS)	#CS Waiting at BAC
	Berthing Area Container (BAC)	#CS Waiting at Harbor
		#CS Loading
		# CS Berthing

Harbor Control	Tugboat (TB)	
	Tugboat Refueling Station	

**Table 4:** Berthing activity definition.

Assumptions	Confidence	Impact
Tugboats do not move empty if both operate at same port	Medium	Low
Ship arrive with respect to a Poisson process	High	Medium

Simplifications	Confidence	Impact
Loading time is independent from ship size	High	Medium
