# Supplementary material for: A conceptual modeling framework for discrete event simulation using hierarchical control structures
Source: Simul Model Pract Theory. 2015 Aug;56:82–96. doi: 10.1016/j.simpat.2015.04.004 (PMC4687135; doi:10.1016/j.simpat.2015.04.004)
Supplement: Supplementary data 1 [file mmc1.pdf]

Appendix A. The Complete Conceptual Model

In the following a summary of the complete conceptual model for the port problem is given.

Appendix A.1. Problem Description

The problem consists of two ports, one for tankers and one for container ships. In both ports tugboats operate to berth and de-berth ships. The main sequence of activities can be described as follows. Tankers and container ships arrive at a corresponding harbor site with respect to an arrival process, where they wait to be towed to a loading area by a tugboat. In the loading area they are berthed by the tugboat and then filled with oil. After the loading process, they are de-berthed and towed to the harbor, also by tugboats, as soon as tugboats are available, where they leave the model. Both berthing and de-berthing, are performed in a FIFO way according to the arriving and loading completion times of tankers. The berthing area holds a certain number of actual berthing sites.

Further, tugboats have to be re-fueled when their fuel level reaches a lower limit. Tugboats can move between the harbor area and berthing area in the same port with no ship in tow ("moving empty") under the following conditions:

- A tugboat moves from a berthing area to the harbor area in the same port if:

    1. no ship is ready for de-berthing;
    2. at least one ship is waiting in the harbor; and

3. a berthing site is available.

- A tugboat moves from the harbor to the berthing area in the same port if:
  1. no ships are waiting in the harbor; and
  2. at least one ship is loading or waiting for de-berthing.
- If both tugboats are working in the same port tugboats are not allowed to move without a ship in tow. This condition assumes that arrivals and loading completions are somewhat balanced, so the two tugboats will be spread evenly "enough" between the harbor and berthing area while performing towing duties. It also keeps the rules for controlling tugboats at a degree of complexity that is not beyond the scope of this paper.

A port reports that it is idle if no ships are present and the tugboat is waiting in the harbor area. The tugboat is sent to the other port if at least three ships are waiting in the other harbor area. Tugboats can only move between the harbor areas of the two ports. Further, a port requests a tugboat back if a ship arrives and waits in the harbor area. If the fuel level of a tugboat reaches a lower limit the tugboat moves to the tugboat service station where it is served according to the FIFO principle. After completion of the refueling service, the tugboat is sent to the port with less tugboats unless the port with less tugboats is empty and the other port is not.

Appendix A.2. Objectives

The objectives of the simulation study can be divided into the organizational aim, general and modeling objectives. They are summarized by table A.5.

Appendix A.3. Outputs

The proposed output measures are summarized by table A.6.

Appendix A.4. Input Factors

Input factors are summarized by table A.7.

Appendix A.5. The Model Structure

The model structure is summarized by the entity structure and the structural blueprint of the model, illustrated by figures A.9 and A.10.

Appendix A.6. Individual Model Behavior

The individual model behavior is summarized by the flow diagrams of active entities, see figure A.11 and detailed activity descriptions, see tables A.8 to A.12. Note that only definitions of tanker activities are provided, as container ships behave analogously.

Appendix A.7. System Behavior

The description of the system behavior is given by the control tree, see figure A.14, control unit specifications, see table A.13, and control policies, see figures A.13 and A.12. Definitions for the container ship port can be defined analogously.

Appendix A.8. Simplifications and Assumptions

Simplifications and assumptions made during the modeling process are summarized by table A.14.

Organizational Aim
Throughput times of ships should be less than 6 hours for 90 % of ships

General Objectives	
**Project Duration**	4 months
**Workload**	2 Developers for a total of ten man weeks
**Run Time Requirements**	Simulation runs should not take longer than 10 seconds at highest speed
**Visualization Requirements**	A 3D visualization is required for stakeholder presentations, results should include graphical representations
**Reusability Requirements**	The model should be designed with respect to the possibility of future additional clients
**Documentation and Usability**	The simulation tool should be easy to use for non simulation-experts as port managers to conduct future additional experiments. Therefore, a detailed documentation and user guide is required.

Modeling Objectives	
**Current Requirements**	Estimate the number of berthing sites required based on the current number of ships arriving at the port
**Future Requirements**	Estimating the number of berthing sites required if the demand increases in the future, several scenarios have to be evaluated
**Policy Improvement**	Evaluation of different values for the threshold on waiting ships before sending tugboat to assist

Table A.5: Simulation Study Objectives

Aggregated Values	
**Mean Throughput Times**	Average time from arrival to leave for tankers and ships
**90% Quantile Throughput Times**	Throughput time that is not exceeded by 90% of ships for tankers and ships
**Mean Waiting Times**	Average time ships wait (sum of waiting in harbor and berthing area) for tankers and ships
**Mean Waiting Times Berthing**	Average time ships wait for berthing for tankers and ships
**Mean Waiting Times De-Berthing**	Average time ships wait for de-berthing for tankers and ships

Empirical Distributions
Automatic generation of empirical distributions of above time measures (throughput, total waiting and separate waiting times) in form of histograms

Table A.6: Model Outputs

Input Factors	
**#MinShipAssisting**	Threshold of waiting ships before tugboats are sent to assist
**Number Berthing Sites**	Number of available berthing sites (for tanker and container port)
**Arrival Streams for Ships**	Parameters for the Poisson processes representing the arrival streams of tankers and container ships

Table A.7: Model Outputs

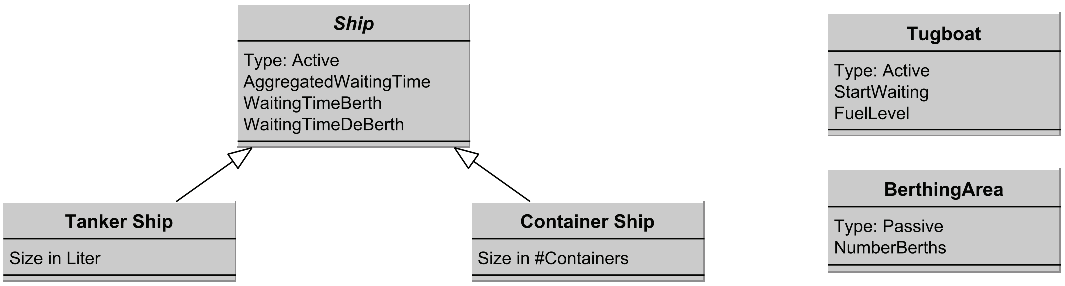

Figure A.9: Entity Structures

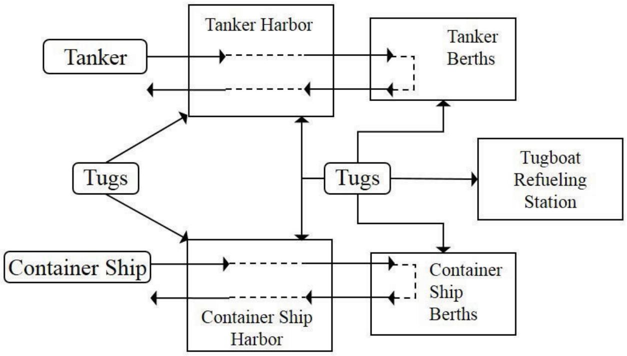

Figure A.10: Structural View of the Port Problem

**Tanker Berthing**	
**Participating Entities**	Tanker , Tugboat (TB)
**Start Type**	Requested
**End Type**	Scheduled
**Start State Changes**	# TA Berthing += 1;
**End State Changes**	TB.FuelLevel = TB.FuelLevel - FuelCons;<br># TA Berthing -= 1;
**Attributes**	**Description/Value**
Duration	20min
FuelCons	10l
**Request Attributes**	**Description/Value**
TimeRequest	When was the request made
**Request Specification**	Request is filed at tanker arrival

Table A.8: Berthing Activity Definition

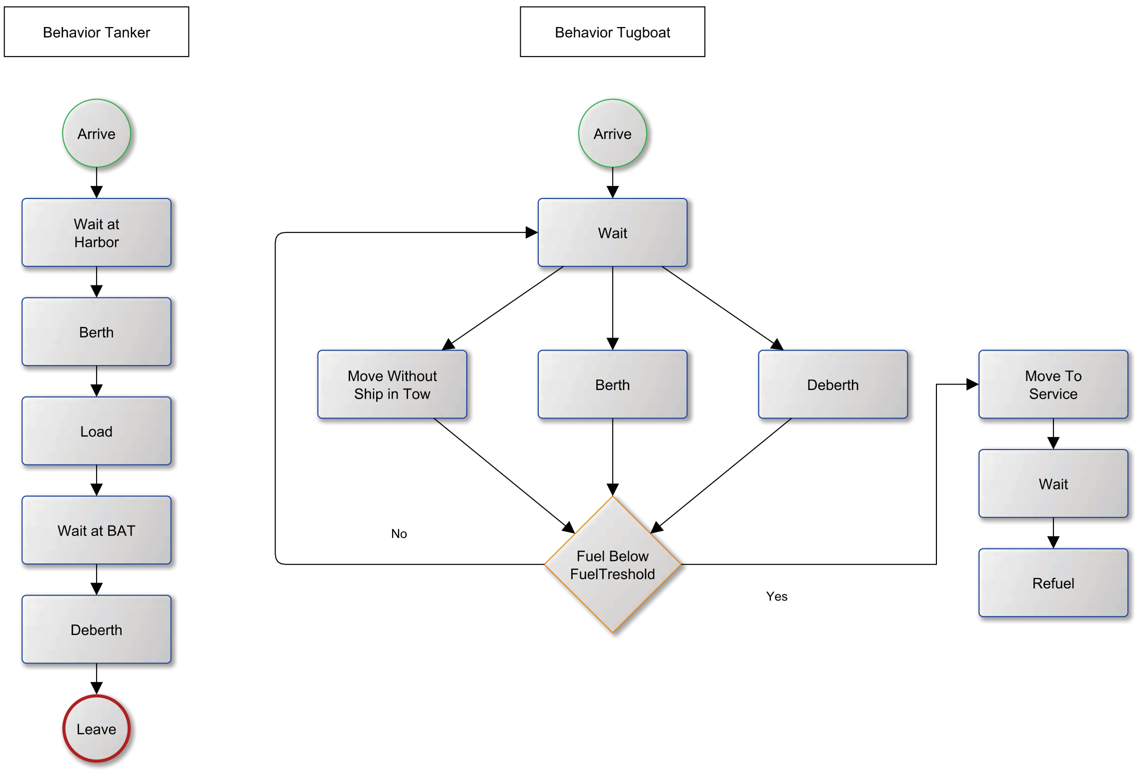

Figure A.11: Behavioral View Port Problem

**Tanker Loading**	
**Participating Entities**	Tanker , Berthing Area Tanker (BAT)
**Start Type**	Sequential
**End Type**	Scheduled
**Start State Changes**	# TA Loading += 1;
**End State Changes**	# TA Loading -= 1;
**Attributes**	**Description/Value**
Duration	2h

Table A.9: Loading Activity Definition

Tanker Waiting	
**Participating Entities**	Tanker, Harbor or Berthing Area Tanker
**Start Type**	Sequential
**End Type**	System
**Start State Changes**	# TA Waiting at BAT (or Harbor) += 1;<br>StartTime = time
**End State Changes**	# TA Waiting at BAT (or Harbor) -= 1;<br>TA.AggregatedWaitingTime += time - StartTime<br>TA.WaitingTimeBerthing = time - StartTime (or de-berthing)
**Attributes**	**Description/Value**
StartTime	Time activity starts

Table A.10: Berthing Activity Definition

Tanker De-Berthing	
**Participating Entities**	Tanker , Tugboat (TB)
**Start Type**	Requested
**End Type**	Scheduled
**Start State Changes**	none
**End State Changes**	TB.FuelLevel = TB.FuelLevel - FuelCons;
**Attributes**	**Description/Value**
Duration	30min
FuelCons	15l
**Request Attributes**	**Description/Value**
TimeRequest	When was the request made
**Request Specification**	Request is filed after tanker loading

Table A.11: De-Berthing Activity Definition

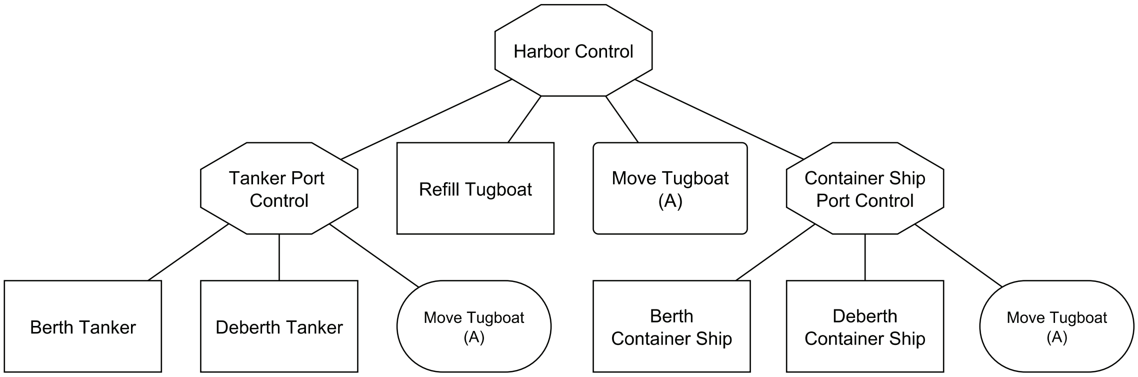

Figure A.12: Control View of Port Problem

**Move Tugboat**	
**Participating Entities**	Tugboat
**Start Type**	System
**End Type**	Scheduled
**Start State Changes**	none
**End State Changes**	TB.FuelLevel = TB.FuelLevel - FuelCons;
**Attributes**	**Description/Value**
StartLocation	Start of move
EndLocation	End of move
Duration	Function of start and end location
FuelCons	15l

Table A.12: De-Berthing Activity Definition

**Control Unit Definition**		
**Name**	**Entities**	**Attributes**
Tanker Port	Tugboat (TB)<br>Tanker Ship<br>Berthing Area Tanker (BAT)	MinShipAssisting = 3<br>#TA Waiting at BAT<br>#TA Waiting at Harbor<br>#TA Loading<br># TA Berthing
Container Port	Tugboat (TB)<br>Container Ship (CS)<br>Berthing Area Container (BAC)	MinShipAssisting = 3<br>#CS Waiting at BAC<br>#CS Waiting at Harbor<br>#CS Loading<br># CS Berthing
Harbor Control	Tugboat (TB)<br>Tugboat Refueling Station	

Table A.13: Control Unit Definition

**Assumptions**	Confidence	Impact
Tugboats do not move empty if both operate at same port	Medium	Low
Ship arrive with respect to a Poisson process	High	Medium
**Simplifications**	Confidence	Impact
Loading time is independent from ship size	High	Medium

Table A.14: Berthing Activity Definition

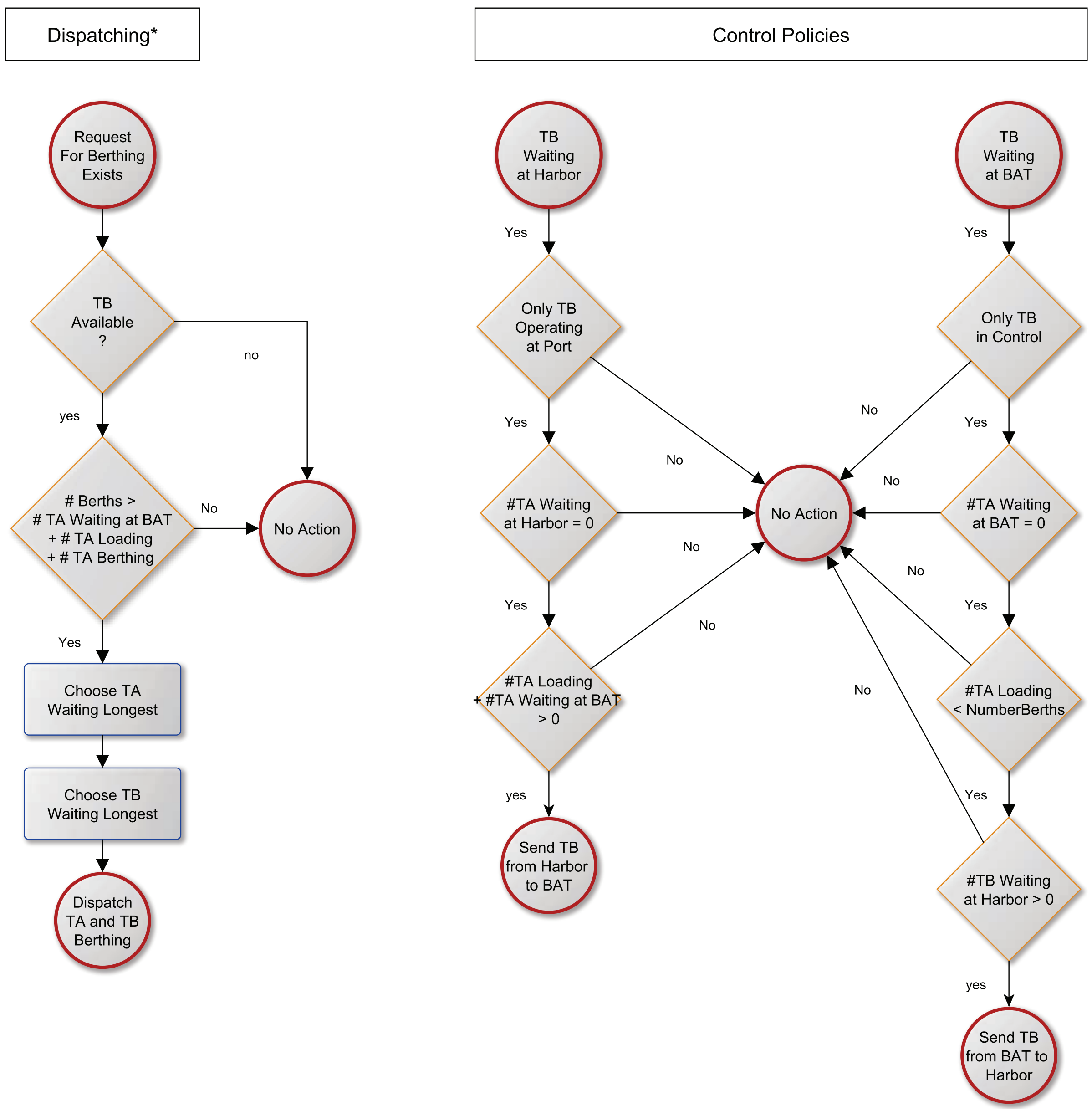

Figure A.13: Tanker Harbor Dispatching and Control Rules (* Only dispatching control rules are shown, but rules for de-berthing can be defined analogously)

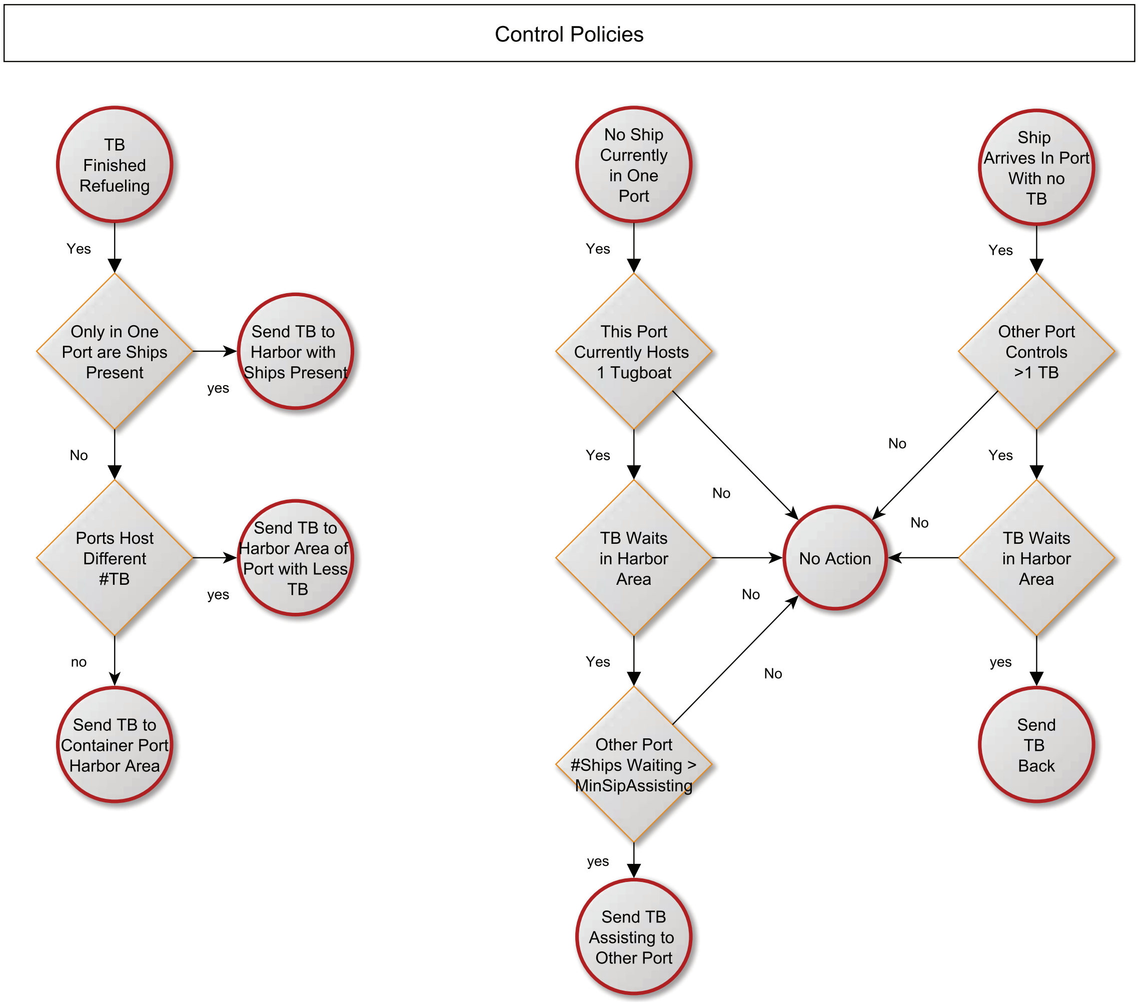

Figure A.14: Overall Port Dispatching and Control Rules
